# High tolerance to temperature and salinity change should enable scleractinian coral *Platygyra acuta* from marginal environments to persist under future climate change

**DOI:** 10.1371/journal.pone.0179423

**Published:** 2017-06-16

**Authors:** Apple Pui Yi Chui, Put Ang

**Affiliations:** Marine Science Laboratory, School of Life Sciences, Chinese University of Hong Kong, Shatin, N.T., Hong Kong SAR, China; Academia Sinica, TAIWAN

## Abstract

With projected changes in the marine environment under global climate change, the effects of single stressors on corals have been relatively well studied. However, more focus should be placed on the interactive effects of multiple stressors if their impacts upon corals are to be assessed more realistically. Elevation of sea surface temperature is projected under global climate change, and future increases in precipitation extremes related to the monsoon are also expected. Thus, the lowering of salinity could become a more common phenomenon and its impact on corals could be significant as extreme precipitation usually occurs during the coral spawning season. Here, we investigated the interactive effects of temperature [24, 27 (ambient), 30, 32°C] and salinity [33 psu (ambient), 30, 26, 22, 18, 14 psu] on larval settlement, post-settlement survival and early growth of the dominant coral *Platygyra acuta* from Hong Kong, a marginal environment for coral growth. The results indicate that elevated temperatures (+3°C and +5°C above ambient) did not have any significant effects on larval settlement success and post-settlement survival for up to 56 days of prolonged exposure. Such thermal tolerance was markedly higher than that reported in the literature for other coral species. Moreover, there was a positive effect of these elevated temperatures in reducing the negative effects of lowered salinity (26 psu) on settlement success. The enhanced settlement success brought about by elevated temperatures, together with the high post-settlement survival recorded up to 44 and 8 days of exposure under +3°C and +5°C ambient respectively, resulted in the overall positive effects of elevated temperatures on recruitment success. These results suggest that projected elevation in temperature over the next century should not pose any major problem for the recruitment success of *P*. *acuta*. The combined effects of higher temperatures and lowered salinity (26 psu) could even be beneficial. Therefore, corals that are currently present in marginal environments like Hong Kong, as exemplified by the dominant *P*. *acuta*, are likely to persist in a warmer and intermittently less saline, future ocean.

## Introduction

Coral populations worldwide are currently facing threats from global climate changes to which they will have to acclimatize, adapt, migrate, or else become extinct [1–2]. While growing attention has focused on the “escape” potential of corals from tropical reefs into temperature refugia, i.e. areas that are currently marginal for coral growth [3–7], the importance of coral communities that are already present in these marginal areas is also increasingly being recognized. It has been suggested that corals in marginal areas may already be well acclimatized to marginal growing conditions by experience-mediated tolerances [8–9]. On the other hand, these corals could have become genetically well adapted to marginal conditions as a result of selection and evolution of different parental lineages and genotypic characters over generations of exposure to environmental extremes [10–11].

Successful sexual recruitment of corals represents an essential process in maintaining coral communities and in facilitating the recovery of coral populations following disturbances [12–13]. It is a process that is crucial to coral reef resilience and may also be a bottleneck for the success of coral species in the future [10, 14–15]. It is therefore important to understand how present and future combinations of environmental stresses may affect any stage (e.g. settlement and post-settlement) of coral recruitment success, especially for corals that are currently present in the marginal areas. Such information is important to better assess the response and possible roles of marginal coral communities in the future under climate change.

Mean sea surface temperature (SST) is projected to increase from 2.6 to 4.8°C by 2100 as a result of climate change [16]. Mounting experimental evidence suggests that increased SST can impact numerous biological and physiological processes of early life stages of corals, including fertilization success [17–18], embryonic development [17–21]; larval survival [20–25], settlement [21–31] and post-settlement survival [25, 27–28]. However, temperature will not be the only environmental factor affected by global climate change. A future increase in the frequency of monsoonal extremes in precipitation is also projected with rising SST [16, 32–33]. Heavy rainfall will likely increase the risk of reduced salinity in near shore waters. Although the effects of temperature stress on the early life stages of coral have been relatively well explored, there is a paucity of data on the effects of salinity stress on these crucial stages [18]. The few studies that quantified the impact of lowered salinity on corals pointed to relatively comparable effects, including reduced fertilization success [18, 34–37], increased developmental abnormality [18, 20, 35, 37], and increased pre- and post-settlement mortality [38]. Given that mass spawning events have been reported to occur during the start of monsoon season in many localities (e.g. in Guam, Okinawa [34], French Polynesia [35] or Hong Kong [18, 20]), responses of coral recruitment stages to low salinity levels are ecologically relevant and likely to be critical to the persistence of coastal coral populations.

Responses to multiple stressors are more complex than those elicited under single stress scenarios, and often present an entirely new perspective on the capacity of organisms to respond [39]. For example, lowered salinity increases the abnormal embryonic development of coral [18, 20]; lowered temperature exacerbates this increase (synergistic effect) [20] whereas elevated temperature reverses it (antagonistic effect) [18]. Such a complex interplay of tolerance often makes the consequences of environmental changes unpredictable. Significant research efforts have already been extended to evaluate the independent effects of temperature and salinity on early life stages of coral. However, as lowering of salinity would be associated with elevated temperature under global climate change, more focus is needed on the interactive effects of temperature and salinity change if their impacts on corals are to be assessed more realistically.

This present study therefore aimed to evaluate the effects of temperature, salinity and the combination of both factors on settlement and post-settlement phases of *Platygyra acuta*, a dominant broadcast-spawning coral in Hong Kong [40]. This massive scleractinian coral is of high ecological significance and functions as an important reef-builder that shapes the coral community structure in Hong Kong and nearby regions [40, 41]. Experimental evidences suggest that early life stages of *P*. *acuta* from Hong Kong are tolerant to environmental stresses. Examples of these include the high tolerance of fertilization, larval survival, motility, settlement and growth to heavy metal and elevated temperature [42, 43], of fertilization, embryonic development, larval survival, motility and settlement to inorganic nutrients [44], and high tolerance of fertilization success and embryonic development to reduced salinity and elevated temperature [18, 20]. However, the effects of salinity and temperature stresses on later stages (i.e., settlement, post-settlement survival and early growth) of the corals have not been thoroughly examined. As Hong Kong is a marginal area for coral growth [40], understanding the responses of Hong Kong corals to environmental change should be of particular significance to the future fate of corals and coral reefs. The extent to which recruitment stages of the dominant species from this region can withstand the interactive effect of temperature and salinity changes points to the potential for this species to persist under a warmer and intermittently less saline future ocean.

## Materials and methods

### Spawning and collection of gametes

This study was conducted in A Ye Wan (AYW), one of the coral core areas in Tung Ping Chau Marine Park (TPCMP) (22^o^32’N, 114^o^25’E), Hong Kong, with permission from the Marine Parks Division of Agriculture, Fisheries and Conservation Department of Hong Kong SAR Government. As described in Chui et al. [45], *P*. *acuta* releases bundles containing eggs and sperm during synchronous multi-specific spawning events in Hong Kong from May to June. Colonies were monitored *in situ* from 19:30 to midnight for the release of bundles, using SCUBA. During the spawning event, egg-sperm bundles from eight individual colonies of *P*. *acuta*, 2–3 m apart at depths of -0.5 to -1.5 m Chart Datum (CD), were collected separately underwater using 150-μm nylon mesh bundle collectors, and then transferred to a makeshift laboratory on shore. Culture procedures followed those in Chui et al. [45] and Omori and Iwao [46]. Briefly, the collected egg-sperm bundles from different colonies were mixed in a fertilization tank and left for an hour to allow for fertilization. Subsequently, the fertilized eggs (embryos) were washed three times to remove excess sperm before being allocated in different culture containers with 40 μm filtered seawater (FSW). The embryos and the resulting lecithotrophic (i.e., energy-limited) larvae were reared at a density of around 0.5 individual/ml. Seawater was changed every day to keep the larvae healthy until being transferred to the Marine Science Laboratory, The Chinese University of Hong Kong, four days after spawning. Larvae brought back were maintained in culture containers filled with freshly prepared 40 μm FSW until the experiment.

### Laboratory experiment

#### Larval settlement

**Fig 1:**
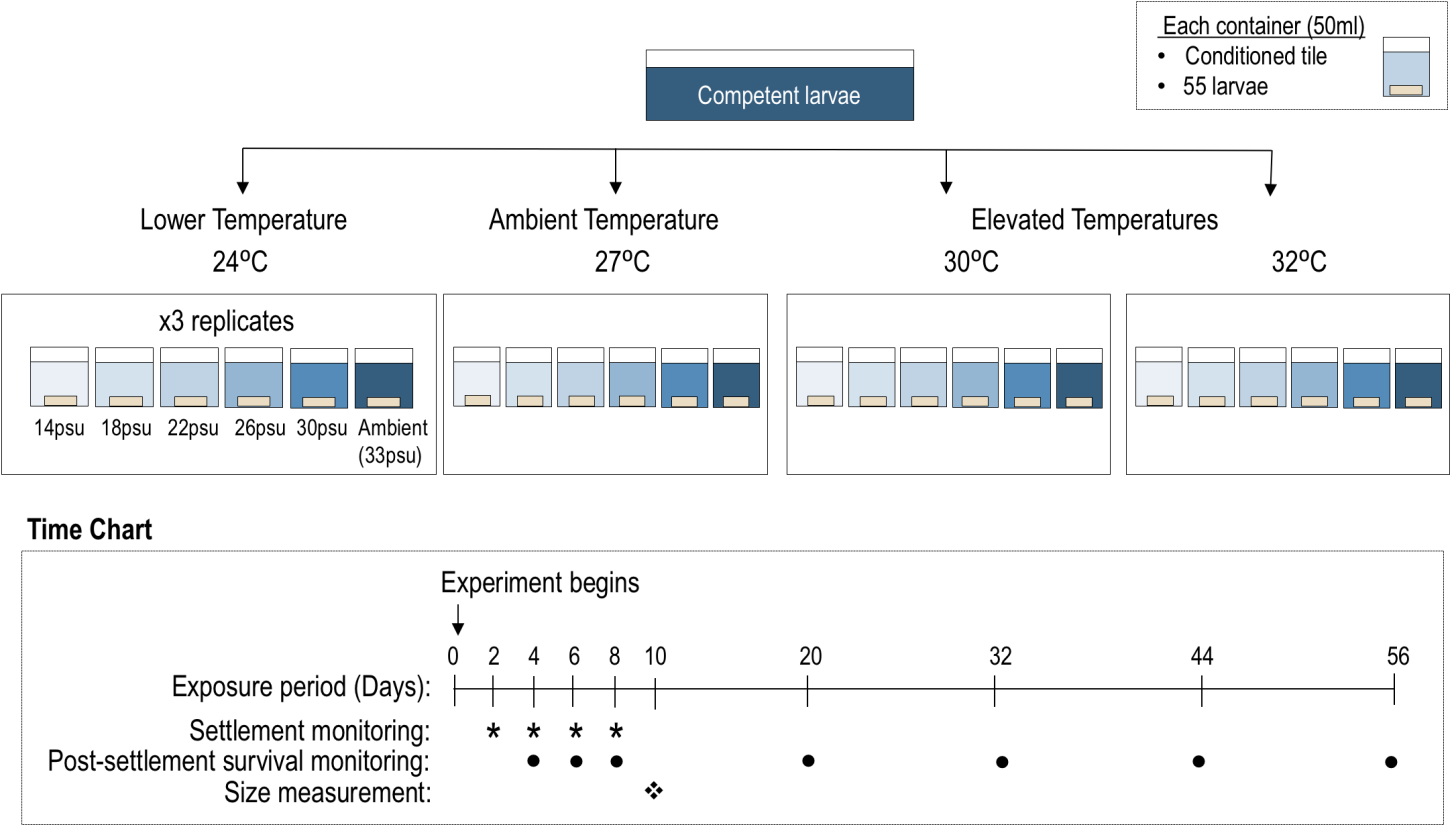
Schematic representation of the experimental design to study the interactive effects of temperature and salinity change on *Platygyra acuta* larvae.

This experiment was conducted six days after spawning, and tested the individual and interactive effects of temperature and salinity on larval settlement of *P*. *acuta*. Experimental design followed those described in Chui and Ang [18]. Briefly, a factorial experimental design (Fig 1) was employed with four levels of temperature (24, ambient 27, 30, 32°C) and six levels of salinity (ambient 33, 30, 26, 22, 18, 14 psu). The temperature range used encompasses current and future projected temperature conditions under global climate change. Temperature of 27°C is the mean ambient temperature of the spawning months in Hong Kong, whereas elevated temperature represents the average summer maximum in the study site (+3°C, i.e. 30°C) as well as an estimated increase in SST by 2100 (+5°C, i.e. 32°C) [16]. All water baths were regulated with chiller and heater to minimize daily temperature fluctuation. HOBO Temperature Pendant data loggers (Onset) were deployed in the water baths to record temperature at 30-min intervals. The levels of salinity employed also represented some realistic ranges recorded in Hong Kong waters. Low salinity ranges are usually recorded in western waters of Hong Kong where the influence of the Pearl River discharge is significant. Low salinity could also be recorded in eastern waters where most corals are found. For example, on May 2014, salinity of 26 psu persisted for more than a day throughout the coastal water column in the study area due to continuous discharge of underground seepage after a heavy downpour [18]. These low salinity ranges therefore represent potential scenarios that could be encountered by corals in Hong Kong.

The desired salinity level was prepared by diluting 0.22 μm FSW with a volume of milli-Q water. Diluted natural seawater was used, rather than artificial seawater, to simulate as closely as possible dilution of seawater by rainfall or underground seepages under natural conditions [35–38]. For control and treatments (each with three replicates), approximately 55 competent larvae were randomly allocated into a beaker containing 50 ml of seawater of specific salinity and a grooved 2.5 x 2.5 cm^2^ ceramic tile that had been preconditioned for 1.5 months *in situ*. Beakers were placed in water baths equipped with thermostats to maintain the respective stable temperatures throughout the experiment. Tiles were examined at 2, 4, 6 and 8 days after treatment exposure (Fig 1). The number of metamorphosed larvae was counted under a dissecting microscope and their locations on the settlement tile were mapped. Metamorphosis was assessed as the percentage of larvae that attached onto the settlement tiles and formed flattened disks of tissue or single polyps. Seawater in each beaker was changed every 48 h with the seawater being pre-conditioned to the specific temperature for each treatment accordingly. Much care was taken not to disturb the larvae during replacement of freshly prepared seawater.

#### Post-settlement survival

Post-settlement survival of recruits was also recorded during the settlement experiment (Fig 1). After eight days of treatment exposure, non-settled larvae were removed. Larvae that settled on the tiles were further monitored every 12 days in the subsequent 48 days, from mid June to early August, under the dissecting microscope for post-settlement survival. Seawater in each beaker was changed every 2–3 days, as described above. Average (± SD) temperature of the water baths during the experimental period was recorded to be 24.1 ± 0.5°C, 27.1 ± 0.5°C, 30.7 ± 0.7°C and 32.7 ± 0.7°C respectively for the four temperature treatments.

#### Size of recruits

The sizes of the post-settled coral recruits were determined by measuring their surface area 10 days after settlement (Fig 1). Only recruits that were marked to have settled since the first two days of the settlement monitoring period were measured to avoid difference in sizes resulting from age difference of these recruits. The cross-sectional area of each individual settled on the horizontal surface was calculated using the image analysis program Image-Pro® Plus 6.0 (Media Cybernetics, Inc., USA). Individuals that settled on the sides of the tile grooves or exhibited partial or full mortality were excluded from the analysis.

As in most broadcasting corals, larvae of *P*. *acuta* are azooxanthellate and do not acquire symbionts in their pre-settlement stages [27, 47–48]. The recruits were infected with *Symbiodinium* after Day 10 (i.e. after size measurement was made) to ensure that differences in recruit sizes were due to treatment effects only.

### Statistical analysis

All statistical analyses were conducted with SPSS version 19.0 for Windows (SPSS Inc., USA). All results are given as mean ± standard error. Proportional data were arcsine transformed and tested for normality using One-Sample Kolmogorov-Smirnov Test and for homogeneity of variances using Levene’s test. A two-factor analysis of variance (ANOVA) was then used to evaluate significant differences (*P*<0.05) of the combined effects of seawater temperature and salinity on the percentage of larvae that settled and the size of recruits. Mixed ANOVA was used to assess the combined effects of seawater temperature, salinity and duration of exposure on post-settlement survival of coral recruits. Post hoc comparisons of means for significant factors in the ANOVAs were carried out using the Tukey Honestly Significant Difference (HSD) test where appropriate. Pairwise comparisons were used to compare the treatment effects of salinity alone at each level of temperature on cumulative percentage of surviving settler and overall post-settlement success. Bonferroni correction to the *P* value was used to adjust the probability of type I error in multiple comparisons.

## Results

### Larval settlement success

**Fig 2:**
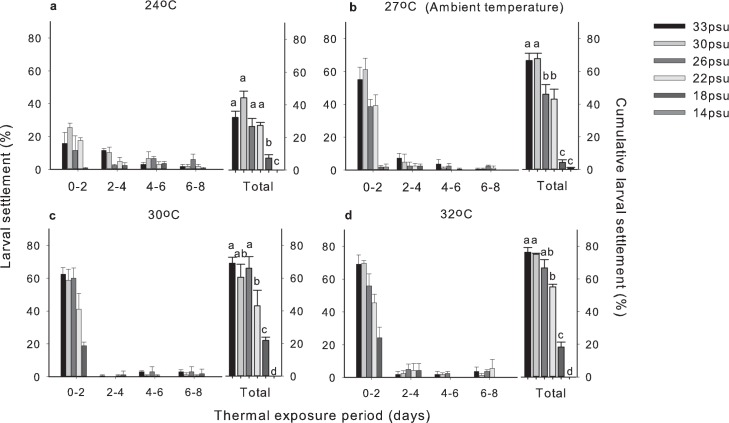
Mean percentage of newly settled *Platygyra acuta* larvae and cumulative surviving settlers. Mean (+SE) percentage (%) of newly and cumulatively settled *Platygyra acuta* larvae in response to different temperature and salinity treatments (*n* = 3 replicates per treatment). Data indicated with the same letter showed no significant difference in settlement success (Two-way ANOVA, *P* > 0.05; Table 1A).

Larval settlement was recorded under all temperature and salinity treatments over the experimental period except at 14 psu, where settlement only occurred under ambient (27°C) temperature (Fig 2). Under ambient and elevated temperatures (30, 32°C), the majority of the larvae (> 80% of total larval settlement) settled in the first two days of the experiment. Within the same time interval, however, the settlement rate was lower (< 60%) under the lowest temperature (24°C) (Fig 2A). All settled larvae survived within this period except those at 18 psu and 14 psu under ambient temperature, as well as at 18 psu under elevated temperatures where mortality was observed. At ambient salinity (33 psu; Fig 2), the total percentage of larval settlement remained consistently high, from 76.4 ± 2.8%, 69.1 ± 3.6% to 66.7 ± 4.4% (mean ± SE) under 32, 30 and 27°C respectively. This significantly declined to 32.1 ± 4.0% under the lowest temperature of 24°C (Pairwise comparisons, *P* < 0.05). Under ambient temperature (27°C; Fig 2B), salinity at ambient 33 psu to 30 psu did not affect total larval settlement. When salinity was reduced to 26 psu, a significant drop in total larval settlement success to 46.1 ± 6.0% was observed and reached the lowest of <5% at 18 psu and 14 psu respectively (Pairwise comparisons, *P* < 0.05).

There was a significant effect of temperature and salinity on percentage of larval settlement (Two-way ANOVA, *P* < 0.001, Table 1A). In general, the percentage of larval settlement significantly decreased with lowered temperature and salinity below 30 psu (Tukey-HSD tests, *P* < 0.05, (32 = 30) > (30 = 27 (ambient)) > 24°C; (33 = 30) > (26 = 22) > 18 > 14 psu; Fig 2). Moreover, there was an interaction between temperature and salinity (*P* < 0.001) with a negative synergistic effect of lowered temperature and lowered salinity that led to a greater settlement failure.

**Table 1:** Results of Two-way (A and C) and Mixed (B) ANOVA showing the effects of different factors and their interaction on (A) cumulative settlement, (B) post-settlement survival of recruits and (C) size changes of the recruits.

**(A) Settlement (%)**				
	**df**	**MS**	***F***	***P***
Temperature	3	852.809	39.200	<0.001
Salinity	5	5094.217	234.162	<0.001
Temperature x Salinity	15	92.463	4.250	<0.001
Error	48	21.755		
**(B) Post-settlement survival (%)**				
Between- Subjects Effects				
Temperature	3	8457.865	75.160	<0.001
Salinity	4	27973.113	248.579	<0.001
Temperature x Salinity	10	1231.383	10.943	<0.001
Error	36	112.532		
Within- Subjects Effects				
Time	4	12635.983	221.720	<0.001
Time x Salinity	16	1170.762	20.543	<0.001
Time x Temperature	12	332.049	5.826	<0.001
Time x Salinity x Temperature	40	311.810	5.471	<0.001
Error	144	56.991		
**(C) Recruit sizes (mm**^**2**^**)**				
Temperature	3	0.237	61.437	<0.001
Salinity	4	0.922	238.760	<0.001
Salinity x temperature	11	0.009	2.319	0.009
Error	540	0.004		

In addition, the decline in larval settlement with lowered salinity did not vary consistently with elevated temperature. Under ambient temperature (27°C; Fig 2B), a significant drop in settlement success to 46% was first observed at 26 psu. Under elevated temperature (i.e., 30°C and 32°C), the first significant drop to <55% was recorded at a lower salinity of 22 psu (Fig 2C and 2D), lower than that under ambient temperature (27°C).

### Post-settlement survival of coral recruits

**Fig 3:**
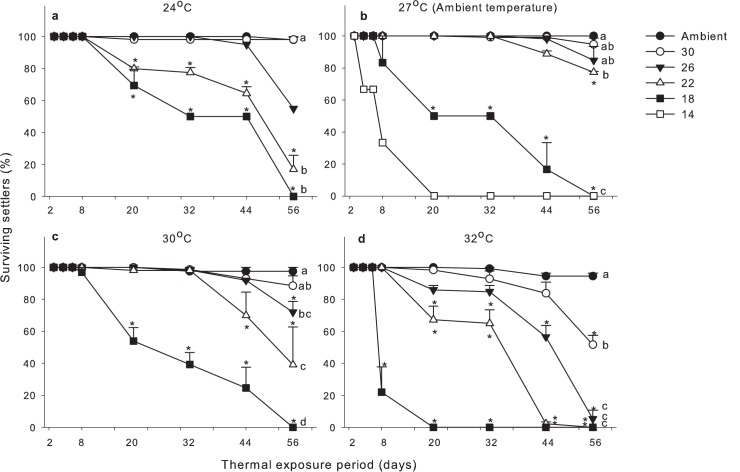
Mean percentage of surviving settlers of *Platygyra acuta*. Mean (+SE) percentage (%) of surviving settlers of *Platygyra acuta* in response to different temperature and salinity treatments (*n* = 3 replicates per treatment). * denotes those results of pairwise comparisons that showed significant difference (*P* < 0.05) between ambient salinity and lowered salinity treatments under the fixed factor of temperature. Data indicated with the same letter showed no significant difference in percentage of post-settlement survival (Mixed ANOVA, *P* > 0.05; Table 1B).

Results of Mixed ANOVA (Table 1B) revealed that post-settlement survival of coral recruits was significantly affected by temperature (*P* < 0.001). Post-settlement survival over the 54 days of monitoring was consistently high (> 94%) across all temperature levels at ambient salinity (33 psu; Fig 3). No mortality of recruits was observed under 27°C; 97.9 ± 2.1%, 97.5 ± 2.5% and 94.6 ± 1.9% (mean ± SE) of the recruits survived under 24, 30 and 32°C respectively. The effect of either lower or higher temperature only began to be detected at lower salinities. Hence, when salinity decreased to 26 psu and below, the percentage of post-settlement survival at ambient 27°C and the elevated temperature of 30°C was always higher than at 24°C, followed by 32°C (Tukey-HSD test, *P* < 0.05, (27 = 30) > 24 > 32°C). Statistics tests on post-settlement survival were not performed for 30 psu and 26 psu treatments at 24°C due to insufficient replicates. Unusually high mortality of recruits under these treatments was recorded, and was likely to be caused by some other external factors, e.g. infectious diseases. Nonetheless, the patterns of post-settlement survival under these two treatments appeared not to be significantly different from those under ambient salinity, at least up to Day 44 of the experiment.

When considered alone, the effect of salinity on post-settlement survival of recruits was also significant (Mixed ANOVA, *P* < 0.001), with higher survivorship consistently recorded at ambient 33 psu (Tukey-HSD test, *P* < 0.05, 33 > 30 > 26 > 22 > 18 psu). None of the recruits survived towards the end of the experimental period at 18 psu across all temperature treatments or at 22 psu under 32°C (Fig 3).

There was a synergistically negative effect between temperature, salinity and duration of exposure (*P* < 0.001; Table 1B) on post-settlement survival of coral recruits. This was revealed by higher mortality in earlier time intervals under the highest elevated (32°C) and lowest (24°C) temperature treatments (Fig 3). The effect was more pronounced at lower salinity levels.

### Size of recruits

**Fig 4:**
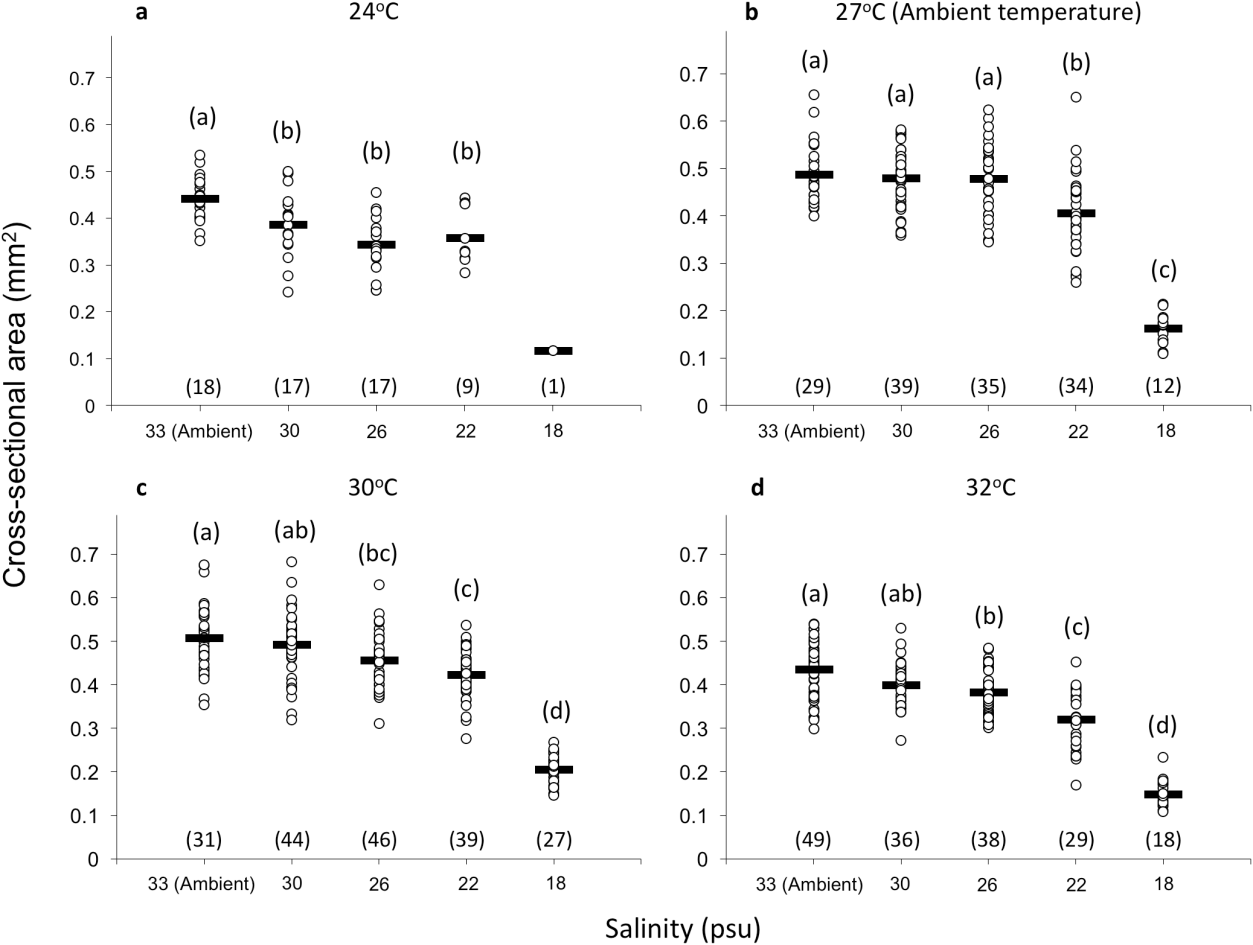
Mean size of *Platygyra acuta* recruits. Mean (+SE) size (mm^2^) of *Platygyra acuta* recruits in response to different temperature and salinity treatments. Number in () under each set of data represents number of replicates. Data indicated with the same letter showed no significant difference in the recruit sizes (Two-way ANOVA, *P* > 0.05; Table 1C).

The size of recruits 10 days after settlement was also affected by temperature and salinity (Two-way ANOVA, P < 0.001, Table 1C). Slightly elevated temperature (30°C) exerted no significant effect on the size of recruits under all salinity levels. However, significantly smaller size of recruits was detected under elevated (32°C) temperature at all salinity levels, except at 18 psu. Lowered temperature significantly reduced the size of recruits under 30 and 26 psu (Tukey-HSD tests, *P* < 0.05, (27 (ambient) = 30) > (24 = 32)°C; (33 = 30) > (30 = 26) > 22 > 18 psu; Fig 4). In addition, there was an interaction between temperature and salinity (*P* < 0.01) with a negative synergistic effect between elevated temperature and lowered salinity that significantly reduced the recruit size (Fig 4, Table 1C). Hence, the size of recruits was significantly reduced at 22 psu under ambient temperature. A similar effect occurred at higher salinity (i.e. 26 psu) under elevated temperatures.

## Discussion

### High tolerance in settlement and post-settlement survival of coral recruits to elevated temperature (+5°C ambient) for up to 56 days of exposure

Results of settlement and post-settlement experiments demonstrate that the effects of near-future rising SST scenarios on *P*. *acuta* may not be as serious as that generally reported or projected in the literature for other tropical coral species. Settlement and post-settlement survivorship of this species were robust to elevated temperature change within the range examined in the present experiments. Although there was an apparent trend of increasing settlement from ambient 27°C (66%) to elevated temperatures of 30°C (+3°C ambient, 69%) and 32°C (+5°C ambient, 76%), the difference was not statistically significant. However, a step-wise response was observed in the lowered temperature of 24°C (-3°C ambient), with a significant drop in settlement to 32%. The settlement monitoring lasted for eight days and the majority of the larvae settled within the first two days of the experiments.

Previous studies have found that if the exposure of elevated temperature is short-term, i.e., from minutes to hours, it can lead to greater larval settlement [27–28]. The trend in prolonged exposure from day to weeks, however, is inconsistent and the results were difficult to interpret. Prolonged exposure to 2 to 3°C above ambient has been found to cause an increase [26, 28], decrease [21, 25–26] or no effect [22, 24, 27, 29–31] in larval settlement. When elevated temperature exceeded +3°C ambient, corals either remained unaffected (+4°C ambient [24]; +6°C ambient [27]) or the settlement rate became significantly decreased (+3.5°C ambient [25]; +4°C ambient [22]; +4°C ambient [24]).

There are two exceptional cases reporting positive effects of elevated temperature exceeding +3°C ambient on coral settlement. The brooding species, *Porites astreoids*, for example, increased settlement rates at 33°C (+5°C ambient [23]). However, since no settlement under control conditions was reported in that study, this positive effect may be an artifact of insufficient settlement cues, as no conditioned tiles or crustose coralline algae were provided during the settlement assay. On the other hand, Nozawa and Harrison [27] also reported a significant increase of 9.2% in settlement at 29°C (+6°C ambient) in *Acropora solitaryensis* after 24h of exposure. However, the positive effect was followed by a 90% post-settlement mortality by Day 5. This points to the importance of studying post-settlement survival in order to reveal the true effects of elevated temperature on future coral sustainability.

There is limited research that has monitored post-settlement survival of corals. Among the aforementioned studies, the longest post-settlement monitoring was conducted by Nozawa and Harrison [28] which showed no significant difference in mean mortality of settled *Platygyra daedalea* recruits exposed to 27°C (ambient) and 29°C (+2°C ambient) for 66 days. In our present study, exposure of *P*. *acuta* recruits to up to +5°C above ambient (i.e. 32°C) under ambient salinity (33 psu) for 56 days did not appear to cause any significant failure in settlement and post-settlement survival. This coral species is by far the first to show such high tolerance of its recruits to such a high level of elevated temperature (+5°C ambient) for up to 56 days of exposure, compared to those reported in the literature [21, 27–28].

### Lowered salinity compromises coral settlement success

In general, as salinity was reduced, successful larval settlement also significantly decreased. This was observed in previous studies on *Acropora hyacinthus*, *Favites abdita*, *Platygyra daedalea* and *P*. *sinensis*, where successful settlement of larvae decreased with reduced salinity from 32 psu ambient to 28 psu [49]. However, no effect of reduced salinity (36 psu ambient to 28 psu) on settlement was reported in *Orbicella faceolata* [38]. These variable susceptibilities of corals to salinity stress could be related to different tolerances of coral species [50]. Moreover, it is plausible that local ambient conditions to which the parent colonies are exposed may also influence the response of their offspring [50–51]. These corals may be in different states of acclimatization and adaptation to salinity stresses, particularly in regions that are frequently exposed to low salinity.

In the present study, larval settlement at ambient 33 psu and 30 psu remained high (>66%) under ambient temperature. Although settlement success decreased when salinity was reduced to 26 and 22 psu, the settlement rates were still above 40%. This suggests that *P*. *acuta* from Hong Kong may also be one of the most tolerant species to settle in some of the lowest salinity levels, compared to literature reports [38, 49]. It is important to note, however, that salinity exposure in the present study was initiated only after the coral larvae became competent (around Day 6). This differs from earlier studies by both Vermeij et al. [38] and True [49] in which salinity treatments were carried out earlier during gamete stages. Further study is needed to verify the extent to which prolonged exposure to reduced salinity will affect the subsequent settlement success of different early-developmental stages of corals.

### Positive effect of elevated temperatures (+3 and +5°C ambient) in enhancing larval settlement success under lower salinity (26 psu)

A major finding of this study is the positive effect of elevated temperature (+3 and +5°C ambient) on larval settlement under reduced salinity level (26 psu). Generally, signs of stress were revealed at reduced salinity of 26 psu under ambient temperature, with a significant decline in settlement success. However, similar decline in settlement success first became noticeable only at much lower salinity (22 psu) under elevated temperatures. This was followed by high post-settlement survival for up to 44 days and 8 days under +3°C and +5°C ambient respectively. This observation is significant as it points not only to the importance of understanding the interactive effects of multiple factors but also the importance of duration of exposure to these effects when evaluating the potential consequences of global climate changes on future coral sustainability. The reasons for this mitigation role of elevated temperature remain unclear. Given that mortality of settled coral larvae was generally reduced compared to swimming larvae [38], such increases in coral larval settlement success under stressful conditions may be an escape mechanism [43]. A similar protective effect of elevated temperature (+3°C ambient) has also been reported to enhance normal development of *P*. *acuta* embryos under lowered salinity (26 psu) [18].

### Reduced recruit sizes as an indicator of energetic cost to cope with temperature and salinity stresses

The majority of coral species, like *P*. *acuta*, have azooxanthellate larvae that do not acquire symbionts in their pre-settlement stages [27, 47–48]. They must rely on their lipid reserves to generate energy for settlement and to form their first skeleton [49]. Differences in early recruit sizes may therefore be a good indicator to reveal the energetic cost of coping with temperature and salinity stresses. The observed significantly smaller sizes of recruits under lowered salinity levels, lowered (-3°C ambient) and elevated temperatures (+5°C ambient) are consistent with the hypothesis that resources are more rapidly used up in these corals to cope with environmental stresses than to sustain metamorphosis and calcification [52]. Reduced size can also reduce competitive ability and increase the risk of post-settlement mortality [53].

### Thermal and salinity tolerances of *Platygyra acuta*

Elevated temperatures of +3 and +5°C ambient did not cause any negative effect on settlement and post-settlement survival in *P*. *acuta* over 56 days of prolonged exposure, making this the most tolerant coral species recorded so far in literature. These findings agree with previous studies on the early life stages of *P*. *acuta* from Hong Kong, which have suggested their high tolerance to environmental extremes [18, 42–44]. Possible reasons for the relatively high thermal tolerance of corals may be related to a large annual range of mean seawater temperature in Hong Kong, from 16°C in winter to 30°C in summer [18, 20]. Corals may have already been well acclimatized/adapted to temperature perturbation through phenotypic plasticity [8–9] or different parental lineage and genotypic characters that have been selected and evolved over years [10–11]. Range of temperature variation has also been suggested to contribute to latitudinal difference in thermal thresholds in coral fertilization and development [10, 54–55]. These studies provide evidence suggesting there is a lower thermal threshold of coral early life stages at lower latitude tropics than at higher latitude subtropics [54–55]. The high thermal tolerance in *P*. *acuta* in the present study appeared to support this hypothesis. However, the strong tolerance to +5°C ambient (+2°C of summer maxima) in settlement and post-settlement survival for up to 56 days of exposure was unexpected, as this temperature is much higher than what the adult colonies may experience throughout the year.

In addition, corals in Hong Kong are mainly found in shallow water regions, from 0 to -3 m CD. They are therefore routinely subjected to salinity effects due to storms and heavy rainfall during wet seasons [18, 20]. High tolerance to salinity stress may thus be a result of acclimatization or adaptation due to long history of exposure [18, 20].

Further research is necessary to look into details of genetic difference [11] and the cell-mediated response in individual coral colonies using gene/protein expression profiling [11, 56–58] to reveal the possible underlying mechanisms behind such high thermal tolerance as well as tolerances to other stresses like low salinity. Susceptibility of other coral species, such as *Acropora* spp., at the same study site should also be examined to test whether such level of tolerance is due to the coral species itself or is an adaptive character shared among corals in Hong Kong in response to similar environmental conditions.

In summary, the results of this study suggest that the combination of predicted elevated temperature and the potential episodes of prolonged lowered salinity over the next century [16], may not pose major problems for the recruitment success of *P*. *acuta*. The combined effect may even be beneficial in certain scenarios, given that the exposure duration is within the tolerance ranges of the coral. *P*. *acuta* is one of the most dominant scleractinian coral species in marginal environments, such as Hong Kong [41]. Further research is necessary to determine whether similar tolerances of the early life stages are shared among other corals in Hong Kong and to identify the underlying mechanisms. Other physical and biological factors may be considered in these studies, including the influence of symbionts on settlement, post-settlement survival and recruit growth rate. These coral species have long pre-empted the space and shaped the community structure in these non-reefal communities [41]. If they are unlikely to be significantly affected by climate change in the near future, the communities dominated by them will likely persist and have important roles to play in maintaining the survival of world’s coral reefs in a warmer and potentially less saline future ocean.
